# Basal androgen status as a modifiable predictor of poor ovarian response in controlled ovarian hyperstimulation

**DOI:** 10.3389/frph.2026.1804297

**Published:** 2026-04-22

**Authors:** M. L. Münch, U. Bender, A. Marshall, T. Guezel, K. Spaich, A. Germeyer, M. Krivega, T. Strowitzki, E. Capp, J. Rehnitz

**Affiliations:** 1 Department of Gynecologic Endocrinology and Fertility Disorders, University Hospital Heidelberg, Heidelberg, Germany; 2 Department of Obstetrics and Gynecology, Medicine School, Universidade Federal do Rio Grande do Sul, Porto Alegre, Brazil

**Keywords:** androgen levels, DHEA supplementation, ovarian reserve markers, poor ovarian response, testosterone supplementation

## Abstract

**Introduction:**

Ovarian response to controlled ovarian hyperstimulation (COH) is commonly predicted by age, ovarian reserve markers, and outcomes of previous stimulation cycles. Androgens play a key role in follicular recruitment, development, and atresia. This study aimed to evaluate circulating androgens as additional, potentially modifiable prognostic markers of ovarian response.

**Materials and methods:**

We conducted a retrospective cohort study analyzing oocyte yield after COH in relation to early follicular phase androgen levels. Expected ovarian response was classified according to the Bologna criteria. Odds ratios (ORs) for androgen levels and oocyte yield were calculated, and receiver operating characteristic (ROC) curves were generated to assess the predictive accuracy of androgen levels for normal versus poor ovarian response (NOR/POR).

**Results:**

A total of 299 stimulation cycles were analyzed (216 NOR and 83 POR). The POR group was characterized by significantly higher median age, lower anti-Müllerian hormone (AMH) levels, reduced oocyte yield, and significantly lower androgen concentrations. A significant positive correlation was observed between the number of retrieved oocytes and both AMH and testosterone levels. The odds of retrieving more than three oocytes were increased for testosterone levels >0.21 ng/mL (OR: 2.67) and for DHEAS levels >0.93 ng/mL (OR: 1.37). While AMH demonstrated the strongest predictive performance for an unfavorable outcome (oocyte yield ≤3; AUC: 0.877), androgen levels showed a moderate predictive ability for poor ovarian response (testosterone AUC: 0.659; DHEAS AUC: 0.610).

**Discussion:**

Considering the observed association between androgen levels and ovarian response, the assessment of androgens prior to COH may provide additional prognostic value. Androgens could represent modifiable biomarkers to identify patients who may benefit from pre-treatment androgen supplementation in the presence of low baseline levels.

## 1 Introduction

The ovarian response to controlled ovarian hyperstimulation (COH) via *in vitro* fertilization (IVF) is crucial for the success of reproductive techniques, as the likelihood of obtaining a euploid blastocyst depends primarily on the number of retrieved oocytes (1, 2). Inadequate response to COH, titled “poor ovarian response” (POR), remains a major problem in reproductive medicine. Many approaches to address unfavorable outcomes after COH have failed because of inconsistencies in the definition of ovarian response.

To standardize the definition of POR and create a prognostic tool for ovarian response, the Bologna criteria include age, laboratory or sonographic ovarian reserve markers and a previous history of low ovarian response (3). However, its predictive value is not significant for women younger than 40 years.

In this context, the POSEIDON classification (Patient-Oriented Strategies Encompassing Individualized Oocyte Number) (4) has gained relevance, as it provides a more differentiated view of POR and better predictive power than does Bologna. The division into four groups [expected vs. unexpected POR in the context of age and anti-Mullerian hormone (AMH) or antral follicle count (AFC)] enables a personalized approach to treatment strategies to address the specific challenges of each patient. Although these core markers allow sufficient prediction of the ovarian response, they cannot be modified before cycle initiation to optimize the expected outcome.

Considering the role of androgens in the initiation of primordial follicle recruitment and early follicular development by increasing FSH (follicle-stimulating hormone) receptor expression raises the question of their predictive value for oocyte yield after COH. Testosterone and its adrenal precursor dehydroepiandrosterone (DHEA), respectively, and its sulfated form dehydroepiandrosterone-sulfate (DHEAS), which is easier to measure in the laboratory, increase the number of growing follicles by impacting follicular atresia (15). A physiological reduction in androgen levels is observed in women with diminished ovarian reserve and advanced age. To date, androgens are not used to predict POR in any classification.

In the era of personalized medicine, success in assisted reproductive technology (ART), especially in poor ovarian response, requires proper evaluation of influencing factors, which may indicate an adjustment of treatment strategies (5, 6).

Data concerning the effects of supplementation with either testosterone or DHEA in POR are still heterogeneous, with a trend toward a positive impact on the ovarian response, number of retrieved mature oocytes, fertilization rates, and good-quality embryos. Moreover, in some studies, clinical pregnancy rates (cPRs) and live birth rates (LBRs) seem to be relatively high.

This study aims to shed light on the ability of androgens to predict the ovarian response as a modifiable factor. As a result, knowledge of suboptimal conditions can potentially serve as a recommendation basis for interventions before starting ovarian stimulation.

## 2 Materials and methods

We performed a retrospective cohort study at the Department of Gynecologic Endocrinology and Fertility Disorders, University Hospital Heidelberg. Data sampling was approved by the institutional ethics committee (S-602/2013) and written informed consent was obtained from all participants prior to inclusion in the study in accordance with the Declaration of Helsinki. Women aged 18–45 years were included for analysis from January 2013 to January 2022 and were subjected to COH, resulting in oocyte pick-up. The exclusion criteria were manifested premature ovarian failure [defined as amenorrhea or oligomenorrhea with raised gonadotrophins and low estradiol (7)], cycle cancellation due to missing ovarian response, premature ovulation or other reasons and missing informed consent. Electronic patient files were retrospectively evaluated for serum androgen levels (testosterone and DHEAS), which were drawn between days 2 and 5 of the menstrual cycle before ovarian stimulation, and cycle outcomes. Testosterone concentrations were quantified using an electrochemiluminescence immunoassay (ECLIA) in heparinized plasma, whereas dehydroepiandrosterone sulfate concentrations were determined by liquid chromatography-tandem mass spectrometry (LC-MS/MS) in serum samples. The classification of poor and normal responses was performed according to the Bologna criteria. Of course, the outcome of the cycle to be analyzed was excluded from the definition.

Statistical analysis was performed with IBM SPSS Statistics V 28.0.0.0/Python 3.9.1, and for graphical presentation, GraphPad Prism V10.2.3 was used. Statistical significance was set to *p* < 0.05. The normality of the data was tested via the Shapiro‒Wilk test. Owing to the lack of absolute limits for androgen values for poor ovarian response in contrast to AMH or AFC, the groups were divided into quantiles. Values below the 25th quantile measured in the entire study cohort were considered “low androgen levels”. The statistical comparison among quartile groups was performed via Welch's *t* test, with a significance level of 0.05.

To quantify the association between androgen levels and oocyte yield, the odds ratio was calculated between low and high androgen values and between mature oocytes less than 3 and those more than 3. A 95% confidence interval (CI) was used to determine the statistical significance of the results.

The receiver operating characteristic (ROC) curve was used to assess the ability of androgens to predict POR. An AUC of 0.5 corresponds to a random classification, whereas an AUC of 1.0 indicates a perfect separation of the groups.

The primary outcome of this study was the number of retrieved oocytes after COH in correlation with the serum levels of testosterone and DHEAS. However, our study did not investigate the correlation between androgen levels and cPR or LBR, nor was there a differentiation between stimulation protocols, dosages or trigger forms.

## 3 Main results

A total of 663 cycles were retrospectively analyzed. Complete data concerning androgen levels (testosterone and/or DHEAS) and outcomes for analysis were available in 299 cycles; 216 (72.2%) were scaled in women with expected normal ovarian response, and 83 (27.8%) were scaled in women with expected poor ovarian response according to the Bologna criteria prior to the cycle to be analyzed. Statistical analysis of both subgroups was performed concerning age, body mass index, AMH levels, the number of retrieved oocytes and the basal hormone level. Baseline characteristics are depicted in Table 1.

**Table 1:** Baseline characteristics. The values are expressed as medians with interquartile ranges [Q25; Q75].

Characteristics	Total Cohort (*n* = 299)	Poor ovarian response (*n* = 83)	Normal ovarian response (*n* = 216)	*p*
Age, y	36 [32; 39]	39 [37; 41]	35 [31; 38]	**<0** **.** **001**
BMI, kg/m^2^	22.7 [20.8; 26.2]	22.4 [20.7; 24.5]	22.8 [21.0; 27.3]	0.092
Oocytes, n	7 [4; 13]	3 [2; 6]	10 [6; 15]	**<0**.**001**
FSH, IU/mL	7.6 [6.2; 9.5]	8.7 [7.1; 11.6]	7.2 [6.1; 8.9]	**<0**.**001**
LH, IU/mL	5.3 [3.8; 6.6]	5.4 [3.9; 6.8]	5.3 [3.7; 6.5]	0.539
E2, pg/mL	46.6 [35.4; 60.8]	58.1 [39.5; 82.4]	44.5 [34.6; 55.8]	**<0**.**001**
AMH, ng/dL	1.89 [1.01; 3.57]	0.76 [0.46; 1.15]	2.53 [1.55; 4.22]	**<0**.**001**
DHEAS, ng/mL	1.51 [0.93; 2.17]	1.33 [0.85; 1.55]	1.56 [1.01; 2.35]	**0**.**019**
Testosterone, ng/mL	0.28 [0.21; 0.40]	0.24 [0.15; 0.31]	0.30 [0.22; 0.41]	**<0**.**001**

AMH, anti-Mullerian hormone; BMI, body mass index; DHEAS, dehydroepiandrosterone sulfate; E2, estrogen; FSH, follicle-stimulating hormone; LH, luteinizing hormone; y, years.

Statistical significance is indicated by *p*-values in bold.

The median age in the NOR group was 35 years, whereas that in the POR group was 39 years. POR was associated with significantly higher FSH levels at higher basal estrogen levels. There was no significant difference in body mass indices between the groups. Compared with NOR, women with predefined POR presented with lower AMH and androgen levels, with high significance for AMH and testosterone, respectively. NOR correlates, on average, with 25% higher testosterone and 17% higher DHEAS levels than POR does.

**Figure 1:**
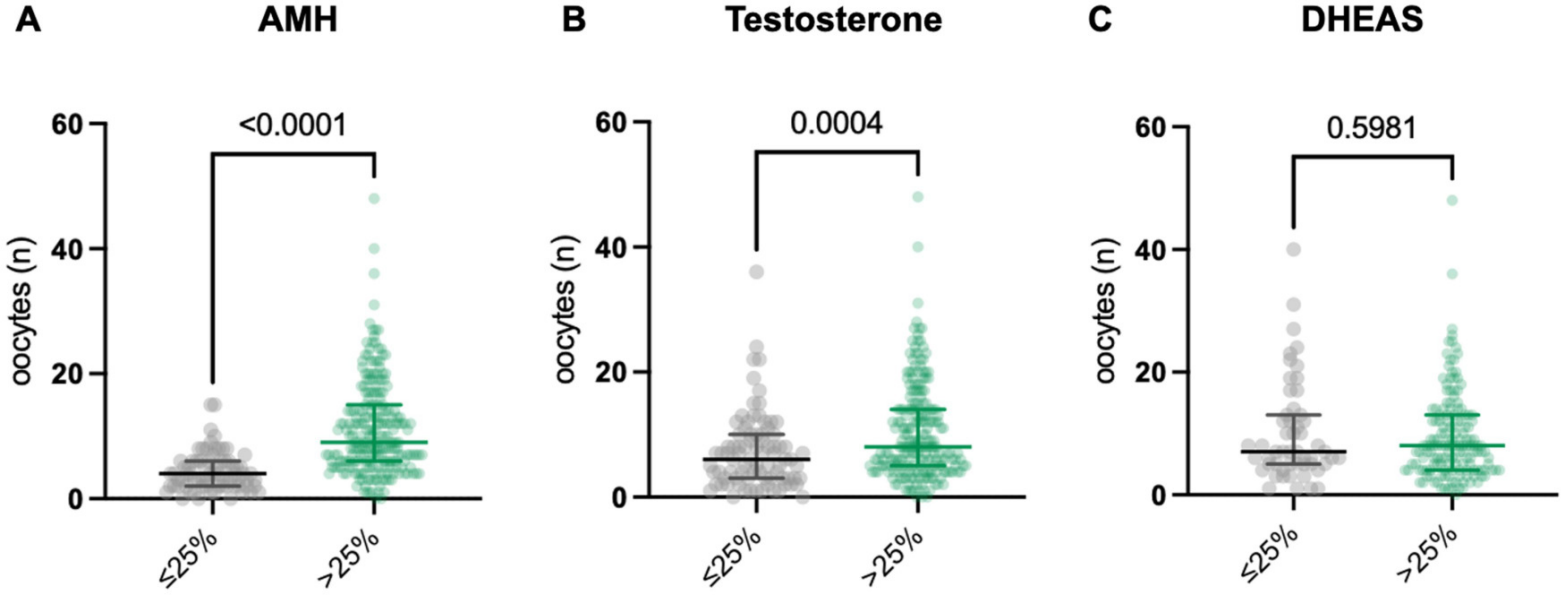
Number of oocytes retrieved on the basis of quantile ranges (≤25th quantile vs. >25th quantile) of AMH **(A)**, DHEAS **(B)** and testosterone **(C)** AMH, anti-Mullerian hormone; DHEAS, dehydroepiandrosterone sulfate.

For the total cohort, we observed a significant positive correlation between the number of retrieved oocytes and AMH (*p* = 0.0001, Figure 1A) and testosterone levels (*p* = 0.0004, Figure 1B). For women with AMH values <1.1 ng/mL or testosterone levels below the 25th quantile of the overall study cohort, significantly fewer oocytes were retrieved after COH.

In contrast, there was a wider range of values for DHEAS, without a significant correlation between the number of oocytes retrieved and the absolute DHEAS level (Figure 1C).

To estimate the event “oocyte count >3 after COH”, the study cohort was predefined in POR and NOR according to the already existing Bologna criteria (especially by AMH/AFC and age). In women with expected POR (AMH levels ≤1.1 ng/mL), those with testosterone levels >the 25th quantile (for our overall cohort, this means values >0.21 ng/mL) were 2.67 times more likely to retrieve >3 oocytes than those with lower values [95% CI: (1.10, 6.48), Supplementary Table 1]. Additionally, patients with DHEAS levels > the 25th quantile (for our overall cohort, this means values > 0.93 ng/mL) have a 37% greater chance of having more than 3 oocytes [OR = 1.37, 95% CI: (0.58, 3.23), Supplementary Table 2]. These findings suggest that even among patients with predicted POR according to a low AMH/AFC or advanced age, higher androgen levels may serve as a useful additional predictor for the actual ovarian response in this subgroup.

**Figure 2:**
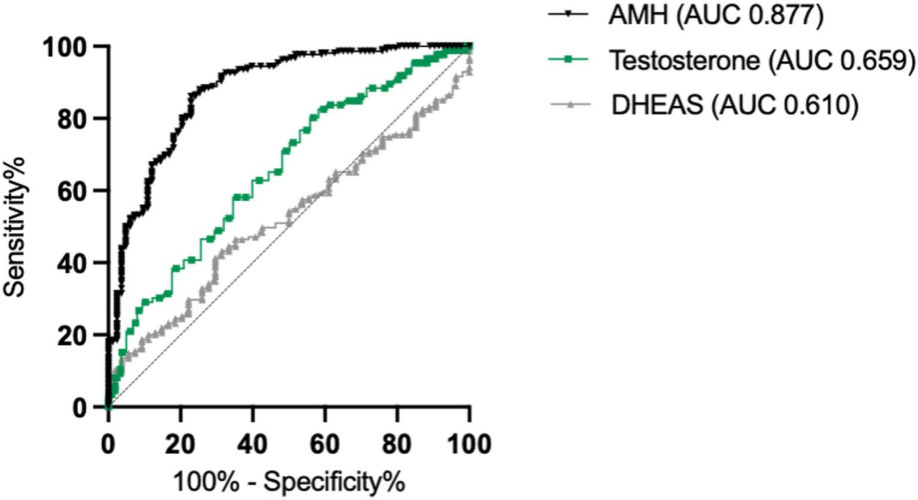
Receiver operating characteristic (ROC) curves for AMH, testosterone, and DHEAS for the total cohort, illustrating their ability to predict oocyte count ≤3 and oocyte count >3. The diagonal line represents the no-discrimination reference (AUC = 0.5). AMH, anti-Mullerian hormone; DHEAS, dehydroepiandrosterone sulfate.

**Figure 3:**
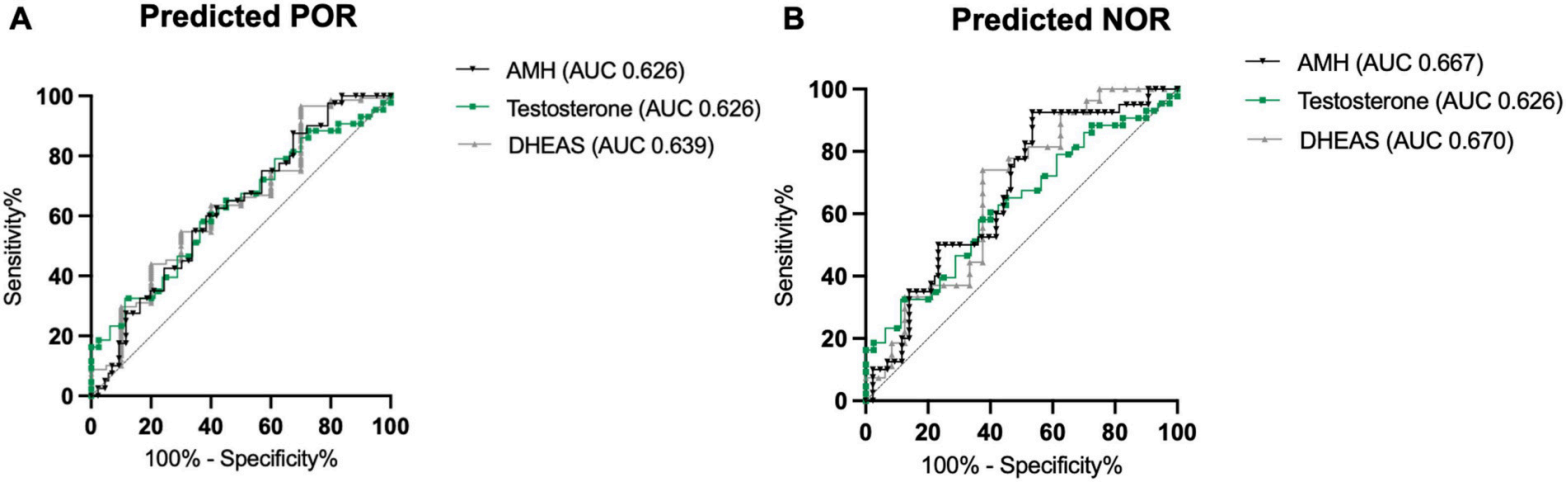
Receiver operating characteristic (ROC) curves for AMH, testosterone, and DHEAS for POR **(A)** and NOR **(B)**, illustrating their ability to predict oocyte count ≤3 and oocyte count >3. The diagonal line represents the no-discrimination reference (AUC = 0.5). AMH, anti-Mullerian hormone; DHEAS, dehydroepiandrosterone sulfate.

To quantify the predictive power of basal serum testosterone and DHEAS levels, ROC curve analyses were used to predict poor ovarian response, which was defined as the number of oocytes retrieved ≤3. For the total cohort (Figure 2), the area under the curve for testosterone values was 0.659, indicating that basal testosterone serum levels presented a moderate but better performance in predicting either a poor or normal ovarian response than did basal DHEAS serum levels (AUC: 0.61). As expected, AMH levels demonstrated the best predictive performance for unfavorable oocyte yield, with an AUC of 0.877. In the subgroup analysis of predefined POR (Figure 3A) vs. NOR (Figure 3B) before the analyzed cycle according to preexisting Bologna criteria, the predictive power was decreased for AMH and testosterone but slightly increased for DHEAS values.

## 4 Discussion

### 4.1 Predicting ovarian response

Poor ovarian response and diminished ovarian reserve (DOR) remain the most challenging problems in clinical practice in the field of ART. The incidence of POR varies from 9% to 24% among patients undergoing ovarian stimulation for *in vitro* fertilization or intracytoplasmic sperm injection (ICSI) (8). As the success of ART is particularly influenced by the number of euploid blastocysts after COH, which can be predicted above all by maternal age (and thus oocyte quality) and ovarian reserve markers, poor responders experience lower implantation rates (2%–4%) with higher cycle cancellation rates (20%) (9).

In terms of individualized medicine, “ART Calculators” attempt to estimate the minimum number of metaphase II (MII) oocytes required to have at least one euploid blastocyst for transfer in patients undergoing COH, leading to tailored treatment strategies to shorten the time to pregnancy (10).

To predict the ovarian response to gonadotropin stimulation, markers, such as anti-Mullerian hormone levels and the antral follicle count, are established tools for counseling patients concerning their prognosis in ART.

The ESHRE consensus for defining poor ovarian response is set out by the Bologna criteria, which include advanced maternal age ≥40 years, previous POR, and an abnormal ovarian reserve test (AFC <5–7 follicles or AMH <0.5–1.1 ng/mL). Two episodes of POR after maximal stimulation are also sufficient to define a patient as a poor responder even without the presence of the aforementioned aspects (3). Despite the predictive ability of these criteria, their performance in cases of unexpected poor response, e.g., in women younger than 35 years or with favorable ovarian biomarkers, is still suboptimal (11–13).

The new POSEIDON criteria aim for a shift from the terminology of poor ovarian response to the concept of low prognosis by taking the individual number of oocytes needed for LBR into account. Female age remains the critical element in the POSEIDON classification because it is crucially related to embryo ploidy and LBR. POSEIDON groups 1 and 3 (aged <35 years) have a low risk of aneuploidy, consequently requiring fewer oocytes to achieve a pregnancy than groups 2 and 4 (>35 years).

However, by definition, the term POR refers to the ovarian response; therefore, at least one stimulated cycle is considered essential for the diagnosis.

The ability to predict the prognosis of ART is one aspect; the other aspect is the modification of unfavorable conditions to improve it. Obviously, age is a nonmodifiable parameter, and ovarian reserve markers cannot be positively influenced. Consequently, the only approach to improve the cumulative LBR in this group of patients is to influence the stimulation response.

In recent years, various strategies addressing the outcome of COH have increased the LBR for POR. Despite a lack of robust data, unfortunately, the holy grail in dealing with POR still does not exist.

### 4.2 The role of androgens in the ovarian response

The metabolic precursor dehydroepiandrosterone, its sulfonated form dehydroepiandrosterone sulfate, which mostly originates from the adrenal glands (80%) rather than ovarian theca cells (20%), is converted to testosterone and dihydrotestosterone in the targeted organs. Both testosterone and DHEA/DHEAS are believed to be involved in early folliculogenesis by stimulating granulosa cell proliferation, increasing the responsiveness to FSH and increasing the recruitment of preantral and antral follicles (14–16); however, follicular atresia is reduced (17–19). In animal models, androgens also affect implantation by increasing the expression of decidualization and endometrial receptivity markers (20–23).

Another interesting link between adrenal and ovarian androgen production and folliculogenesis is depicted by Gleicher et al. in women with primary ovarian failure (POI). A premature reduction in ovarian function is classically associated with hypoandrogenism, primarily as a consequence of insufficient testosterone production in ovarian theca cells. This condition leads to the arrest of small follicle stages, a decrease in granulosa cell mass and estradiol production, a compensatory increase in gonadotrophin levels, and a decrease in AMH levels. However, adrenal hypoandrogenism can also mimic the classical POI phenotype via these mechanisms as a type of secondary ovarian insufficiency (SOI). In cases of SOI, supplementation with androgens may restore ovarian function and normalize responses to pharmacological stimulation (24).

### 4.3 The predictive value of androgens in the ovarian response

In our retrospective analysis, both testosterone and DHEAS levels positively correlated with the ovarian response and the likelihood of achieving more than 3 oocytes after COH. However, while age and AMH levels have strong potential for their predictive ability, androgen levels also seem to have prognostic ability, with moderately robust results in our cohort.

In contrast, previous multivariate models with a smaller sample size of 207 women revealed no significant difference in DHEAS concentrations between poor and normal responders, unlike testosterone, which has very limited ability as a single predictor (25). Some analyses revealed a negative correlation between serum testosterone levels and the total dose of gonadotrophins used during COH, the number of oocytes retrieved, and the number of normally fertilized oocytes but no significant correlation with pregnancy, live birth, or miscarriage rate. Conversely, high androgen levels also do not significantly increase the LBR in infertile women undergoing IVF (26).

### 4.4 Androgen supplementation—a spark on the way to success?

To our knowledge, this is the first study evaluating the predictive value of androgen levels for poor ovarian response that is complementary to the Bologna criteria.

There is currently inconsistent evidence that the adjuvant use of testosterone or DHEA before and during ovarian stimulation improves the ovarian response in terms of ongoing pregnancy rates and live births in poor responders. Heterogeneity is created by a broad variation in defining POR, baseline characteristics of patients, stimulation protocols and the administration of androgen supplements.

DHEA/DHEAS act as mild androgens with lower affinity for androgen receptors than testosterone does. Since androgen levels vary in different organs, DHEA supplementation allows each organ, the ovaries included, to draw organ-specific amounts of precursor for achieving desirable testosterone levels. In contrast, testosterone floods all organs uniformly, overexposing some and underexposing others. Therefore, side effects resulting from direct testosterone administration are consequently more common (27).

Androgen supplementation in POR treatment could facilitate the transition of follicles from dormant to the growing pool during the early and intermediate stages of follicular maturation (28). Casson et al. first reported the beneficial effects of DHEA supplementation in poor ovarian responders (29).

In various subsequent studies and meta-analyses, DHEA pretreatment in POR has been shown to have statistically positive effects on the serum FSH level, total dose of gonadotrophin and number of days of stimulation, estrogen level on the day of human chorionic gonadotrophin (hCG) administration and endometrial thickness. Furthermore, a decrease in miscarriage rates in some trials also suggested a beneficial impact on oocyte quality (30–34).

A non-RCT (randomized controlled trial) by Chen et al. demonstrated a positive association between DHEA supplementation and more than 3 retrieved oocytes (OR = 3.37, 95% CI: 1.64–6.96, *p* = 0.001) in POSEIDON groups 3 and 4, regardless of whether the patients were aged ≤40 or >40 years (35). Compared with the controls, those receiving DHEA had greater numbers of retrieved oocytes, metaphase II oocytes, fertilized oocytes, day 3 embryos and top-quality day 3 embryos.

In addition to the choice of the appropriate androgen dose, the ideal starting point for application before COH cannot be defined according to the existing data. The transition period from preantral to antral follicular stages lasts approximately 70 days in humans (36). As a consequence, the administration of DHEA for at least 3 months is expected to have the maximum effect on the initiation of follicular recruitment (37).

A single-center RCT by Subirá et al. reported the effects of the application of 12.5 mg of testosterone gel per day for 56 and 10 days prior to ovarian stimulation compared with no intervention (38). There was no difference in the number of mature oocytes retrieved or in the pregnancy, live birth, or miscarriage rates among the three groups. These results are largely in hand with those of a similar RCT by Hoang et al., except for a significantly greater endometrial thickness on the day of trigger and significantly higher hCG-positive and ongoing pregnancy rates in the testosterone pretreatment groups (39).

A recently published meta-analysis of 38 RCTs by Conforti et al. summarized the positive effects of DHEA on the number of oocytes retrieved after COH in women with diminished ovarian reserve but not on the number of mature oocytes, cPR or LBR, in contrast to data dealing with testosterone pretreatment (40). Some studies have reported a greater number of high-quality embryos in women supplemented with testosterone. Altogether, 5 RCTs suggested, in addition to an increased oocyte rate, an improved cPR (OR: 2.29) as well as LBR (OR: 2.19). Consequently, to shed more light on the potential benefit of POR, a large RCT must be conducted. In addition to the choice of the appropriate dose, the ideal starting point for application before COH cannot be defined according to the existing data. In human models, it has been suggested that the administration of DHEA for at least 3 months (37) is needed to achieve the maximum effect on the initiation of follicular recruitment. However, this long lead time can be a problem for women with limited time for COH, especially for older patients.

## 5 Conclusions

The inconsistency of data concerning the different treatment strategies for POR needs further identification, and patient characteristics need to be analyzed. Subtle differences in the basic characteristics of women with poor ovarian response could lead to decisive differences in the success of the respective therapeutic approach.

As a potential prediction tool, the measurement of basic androgen levels in combination with the established Bologna or POSEIDON criteria could be considered in counseling couples and planning COH as well as potential additional supplementation. By specifically selecting patients with (adrenal) hypoandrogenism, targeted supplementation could benefit the ovarian response, embryonal development, cPR and LBR in this cohort. To address the controversy in androgen supplementation, further large high-quality RCTs using patients' basic androgen levels as an additional marker for a more explicit selection criterion are needed to reduce heterogeneity and potentially identify patients who would benefit from such a treatment.

### 5.1 Limitations

The results are based on a single-center approach with a limited sample size. Owing to a heterogeneous age distribution and different stimulation protocols, dosages and durations, these aspects were not specifically included in the analysis. Furthermore, in some cases, the current values of the cycle to be analyzed were missing, and the androgen values from the previous cycle had to be used, provided that the interval between blood collection and COH was less than 6 months. Otherwise, this cycle was excluded from the analysis. The data need to be strengthened in subsequent prospective studies with larger and homogenous study groups, including further aspects affecting ovarian response and outcome parameters such as cPR or LBR.

### 5.2 Wider implications of the findings

This analysis depicts a correlation between androgen levels and poor ovarian response. In contrast to age and AMH levels, androgen levels can be modified prior to ovarian stimulation. This, furthermore, supports experimental supplementation, for example, with DHEA or testosterone, particularly in patients with low androgen levels. Consequently, in poor ovarian responders, prestimulatory androgen levels could be used as a potential prognostic tool, in addition to the established and robust markers like AMH levels or AFCs, and might be more diagnostically conclusive than age, potentially leading to the recommendation for supplementing androgens before COH in women with expected POR and low testosterone or DHEAS serum levels, aiming to improve their outcome after ovarian stimulation. By filtering appropriate patients, we hope to achieve more robust and significant treatment effects by adding androgens to individual treatment approaches on the way to pregnancy.

## Data Availability

The raw data supporting the conclusions of this article will be made available by the authors, without undue reservation.
