# Supplementary material for: Basal androgen status as a modifiable predictor of poor ovarian response in controlled ovarian hyperstimulation
Source: Front Reprod Health. 2026 Apr 22;8:1804297. doi: 10.3389/frph.2026.1804297 (PMC13143915; doi:10.3389/frph.2026.1804297)
Supplement: Supplementary file 1 [file Table1.docx]

**Supplementary Tables**

**Supplementary Table 1:**

|  | **Testosterone >0.21 ng/mL** | **Testosterone ≤0.21 ng/mL** |
| --- | --- | --- |
| **Oocytes >3** | 34 | 15 |
| **Oocytes ≤3** | 17 | 20 |

**Supplementary Table 1**: In patients with low AMH (≤1.1 ng/mL), those with testosterone levels >0.21 ng/mL were 2.67 times more likely to retrieve >3 oocytes compared to those with lower testosterone, with a 95% confidence interval of 1.10–6.48. This indicates a significant association between high testosterone and a favorable oocyte yield in this subgroup. These findings suggest that even among patients with low AMH, elevated testosterone may serve as a useful additional predictor of a better ovarian response.

**Supplementary Table 2:**

|  | **DHEAS >0.93 mg/mL** | **DHEAS ≤0.93 ng/mL** |
| --- | --- | --- |
| **Oocytes >3** | 25 | 24 |
| **Oocytes ≤3** | 16 | 21 |

**Supplementary Table 2**: An odds ratio of 1.37 suggests that, among patients with low AMH (≤1.1), those with DHEAS levels above 0.93 have approximately 37% higher odds of retrieving more than 3 oocytes compared to those with lower DHEAS. However, the 95% confidence interval (0.58–3.23) indicates that this difference is not statistically significant. In other words, while there is a trend toward better outcomes in the high-DHEAS group, the data do not provide sufficient evidence to conclude a real effect.
